# Dynamic K-Line Status and Surgical Outcomes in Multilevel Cervical OPLL: A Multicenter Comparative Study

**DOI:** 10.3390/jcm15020520

**Published:** 2026-01-08

**Authors:** Jun Jae Shin, Sun Joon Yoo, Se Jun Park, Dong Kyu Kim, Hyun Jun Jang, Bong Ju Moon, Kyung Hyun Kim, Jeong Yoon Park, Sung Uk Kuh, Dong Kyu Chin, Keun Su Kim, Chang Kyu Lee, Keung Nyun Kim, Tae Woo Kim, Yoon Ha

**Affiliations:** 1 Department of Neurosurgery, Yongin Severance Hospital, Yonsei University College of Medicine, Yongin 16995, Republic of Korea; 2 Department of Neurosurgery, Gangnam Severance Hospital, Yonsei University School of Medicine, Seoul 03722, Republic of Korea; 3 Department of Neurosurgery, Severance Hospital, Yonsei University College of Medicine, Seoul 03722, Republic of Korea; 4 Department of Neurosurgery, Ewha Womans University Seoul Hospital, Ewha Womans University, Seoul 03760, Republic of Korea

**Keywords:** anterior cervical discectomy and fusion, cervical myelopathy, cervical vertebrae, dynamic K-line, laminectomy, laminectomy with fusion, ossification of posterior longitudinal ligament (OPLL)

## Abstract

**Background/Objectives**: To evaluate the clinical and radiological outcomes of surgical interventions stratified by dynamic K-line status and to identify predictors of neurological recovery in multilevel cervical ossification of the posterior longitudinal ligament (OPLL). **Methods**: This study analyzed 535 patients with multilevel cervical OPLL who underwent anterior cervical discectomy and fusion (ACDF), laminoplasty (LP), or laminectomy with fusion (LF), with a minimum 24 months of follow-up. Patients were classified based on dynamic K-line status—neutral (NK-line) and flexion (FK-line)—into three groups: Group 1 (NK-line [+]/FK-line [+]), Group 2 (NK-line [+]/FK-line [−]), and Group 3 (NK-line [−]/FK-line [−]). Radiographic parameters, JOA scores, and VAS were compared, and multivariate regression identified predictors of recovery. A multinomial inverse probability of treatment weighting (IPTW) analysis was conducted to reduce treatment selection bias. **Results**: Progressive dynamic K-line negativity was associated with greater cervical kyphosis, a higher canal-occupying ratio, reduced FK-line distance, and poorer neurological recovery. After IPTW analysis, ACDF showed higher adjusted recovery across subgroups. In Group 1, younger age and fewer operative levels predicted better recovery. In Groups 2 and 3, LF demonstrated significantly greater neurological recovery than LP. A larger preoperative FK-line distance and a greater postoperative FK-line distance increase were independent predictors of favorable outcomes. **Conclusions**: Dynamic K-line stratification has good prognostic value in multilevel cervical OPLL. ACDF remains the most effective procedure across dynamic K-line status groups, and LF is preferred over LP in patients with latent or fixed FK-line (−). Incorporating dynamic K-line metrics into surgical planning could improve procedure selection and enhance neurological recovery.

## 1. Introduction

Multilevel cervical ossification of the posterior longitudinal ligament (OPLL) leads to progressive cervical myelopathy and requires surgical intervention [1,2]. In cases involving kyphotic alignment, extensive ossification, or congenital stenosis, selecting the optimal surgical approach is challenging.

Posterior decompression, including laminoplasty (LP) or laminectomy with fusion (LF), is generally recommended for multilevel OPLL, particularly when three or more segments are involved, due to its ability to address widespread canal stenosis. However, its efficacy can be limited in the presence of cervical kyphosis or significant anterior cord compression, which can restrict adequate posterior cord shift [3]. Alternatively, anterior discectomy and fusion (ACDF), although more direct in alleviating ventral compression, is technically demanding and associated with higher complication rates than posterior options, including dysphagia, cerebrospinal fluid leakage, implant dislodgment, and pseudarthrosis [3].

The K-line, defined as the line connecting the midpoints of the spinal canal at C2 and C7 on neutral lateral radiographs, is widely used to guide surgical decision-making [4]. When the OPLL mass does not cross this line (K-line [+]), posterior decompression is typically effective [4]. In contrast, when the OPLL extends beyond the line (K-line [−]), ACDF is recommended to enable sufficient spinal cord shift [5].

Conventional K-line assessment is static and might not fully reflect cervical dynamics. Recent studies have reported that K-line status can vary with cervical motion. In particular, flexion radiographs can reveal latent anterior compression that is not visible in neutral views [6]. Patients with K-line (+) in the neutral position but K-line (−) during flexion (FK-line [−]) exhibited poor outcomes after LP [6,7]. Those findings highlight the importance of dynamic mechanical factors in the progression and prognosis of the cervical myelopathy associated with OPLL [8,9].

Dynamic radiographs enable patient stratification into three clinically relevant categories: maintained K-line positivity in both the neutral and flexion positions, conversion to K-line negativity during flexion, and persistent K-line negativity in both positions. These dynamic K-line classifications reflect distinct degrees of sagittal imbalance and anterior cord compression, making them clinically relevant predictors of surgical outcomes.

In this retrospective multicenter study, we evaluate the clinical and radiological significance of dynamic K-line status in patients with multilevel cervical OPLL treated with ACDF, LP, or LF. Specifically, we assessed the relationship between cervical alignment parameters and neurological recovery and identified predictors of neurological outcomes based on the dynamic K-line classification.

## 2. Materials and Methods

This retrospective multicenter study reviewed patients diagnosed with cervical OPLL between January 2016 and June 2023 at three university-affiliated hospitals. A total of 648 patients with multilevel OPLL involving three or more vertebral segments underwent ACDF, LP, or LF. Among them, 105 patients were excluded due to insufficient follow-up (n = 54), the absence of initial dynamic radiographs (n = 14), or other confirmed neurological disorders (n = 37), including Parkinson’s disease, cerebral infarction, or hemorrhage. Additionally, 8 patients who underwent combined anterior–posterior decompression procedures were excluded due to the small sample size.

**Figure 1:**
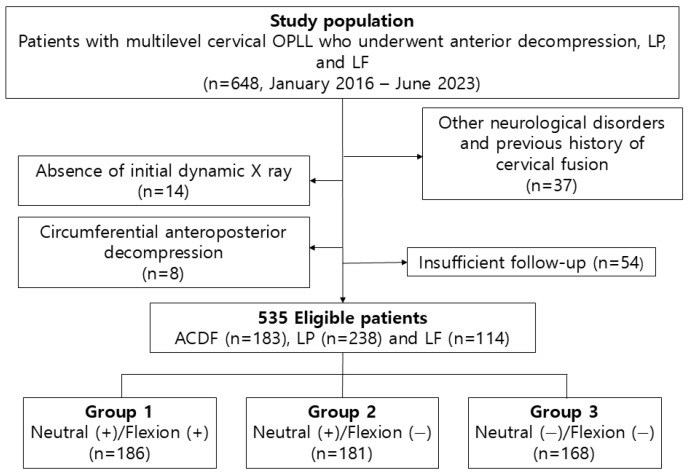
Flow diagram of patient selection and dynamic K-line-based group allocation. Of 648 patients with multilevel cervical ossification of the posterior longitudinal ligament (OPLL) treated between January 2016 and June 2023, 535 patients met the inclusion criteria after predefined exclusions. Eligible patients were stratified into three groups according to neutral and flexion K-line status: Group 1 (neutral [+]/flexion [+]), Group 2 (neutral [+]/flexion [−]), and Group 3 (neutral [−]/flexion [−]).

Ultimately, 535 patients with complete clinical and radiographic data and a minimum follow-up of 24 months were included in our analysis. A flow diagram illustrating patient selection, exclusion criteria, and allocation into dynamic K-line-based groups is provided in Figure 1.

The diagnosis of OPLL was confirmed using plain radiographs and computed tomography (CT). OPLL was classified preoperatively on CT as the segmental, continuous, mixed, or localized type [1]. Cervical myelopathy was diagnosed based on radiographic evidence of cord compression and upper motor neuron signs upon neurological examination. The inclusion criteria were (1) radiologically confirmed OPLL involving three or more vertebral levels; (2) availability of preoperative and postoperative dynamic cervical radiographs; (3) minimum follow-up of 24 months; and (4) availability of complete clinical and radiological data. The exclusion criteria were (1) prior cervical surgery; (2) traumatic spinal cord injury; (3) neoplasm; (4) infection; (5) combined anterior and posterior staged surgery; and (6) other neurological disorders. This study was approved by the Institutional Review Board of Yonsei University Medical Center (No. 9-2025-0143, approved on 28 September 2025) and was conducted in accordance with the Declaration of Helsinki. The IRB waived the requirement for patient consent due to the retrospective design of the study.

### 2.1. Radiologic Evaluation

**Figure 2:**
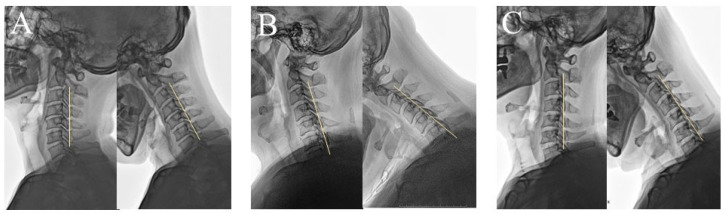
Dynamic K-line classification in multilevel cervical OPLL. Patients were classified into three groups based on dynamic K-line status. (**A**) Group 1: Maintained K-line (+) in both neutral (NK-line) and flexion (FK-line) positions. (**B**) Group 2: NK-line (+) but FK-line (−). (**C**) Group 3: NK-line (−) and FK-line (−). The yellow lines indicate the K line.

In the neutral position, the K-line was drawn by connecting the midpoints of the spinal canal at C2 and C7 on lateral radiographs to determine whether the OPLL mass crossed the line (NK-line [−]) or not (NK-line [+]). Dynamic radiographs obtained in the neck-flexed position were used to assess the flexion K-line (FK-line), classified as FK-line (+) if the OPLL mass did not cross the line and FK-line (−) if it did. The plain lateral radiograph is inverted to facilitate the visualization of the OPLL mass. The patients were categorized into three groups based on their dynamic K-line status: Group 1 maintained K-line (+) in both the neutral (NK-line) and flexion (FK-line) positions; Group 2 had NK-line (+) but FK-line (−); and Group 3 was NK-line (−) and FK-line (−) (Figure 2).

**Figure 3:**
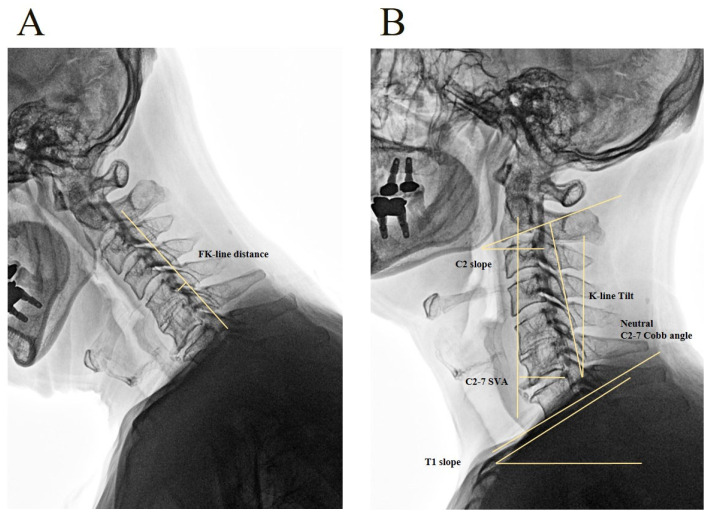
Measurement of radiologic parameters. (**A**) Flexion lateral cervical radiograph (image inverted to enhance OPLL visualization). The flexion K-line (FK-line) distance is defined as the shortest perpendicular distance from the FK-line to the posterior margin of the nearest vertebral body. (**B**) Neutral lateral radiograph demonstrating sagittal parameters: C2 slope (angle between the inferior endplate of C2 and the horizontal plane), T1 slope (angle between the superior endplate of T1 and the horizontal), C2–7 Cobb angle (angle between the inferior endplates of C2 and C7), C2–7 sagittal vertical axis (SVA; horizontal distance from the centroid of C2 to the posterior-superior corner of C7), and K-line tilt (angle between the FK-line and a vertical line perpendicular to the horizontal plane).

The FK-line tilt was defined as the angle between the FK-line and a line perpendicular to the horizon during flexion. The FK-line distance (FKD) was defined as the shortest distance from the FK-line to the posterior border of the vertebral body line on an OPLL lateral neck flexion radiograph (Figure 3A). The change in FK-line distance (ΔFKD) was calculated as the difference between the postoperative and preoperative FKD measurements.

The cervical sagittal alignment parameters were the C2 slope (C2S), the angle between the lower endplate of C2 and the horizontal plane; the C2–7 Cobb angle (CA), the angle between the inferior endplates of C2 and C7; the sagittal vertical axis (SVA), the horizontal distance from the centroid of C2 to the posterior-superior corner of C7; and the T1 slope (T1S), the angle between the upper endplate of T1 and a horizontal line (Figure 3B) [10].

Range of motion (ROM) was defined as the difference between the extension CA and flexion CA. The change in the C2–7 CA (ΔC2–7 CA) after surgery was defined as the postoperative measurement minus the preoperative measurement. The canal-occupying ratio (COR) was calculated as the maximum thickness of the OPLL divided by the anteroposterior diameter of the spinal canal at the most affected level. All radiological measurements were made using PACS software (ZeTTA PACS Viewer version 2.0.2.6, TaeYoung Soft Co., Ltd., Gwacheon, Republic of Korea). Radiological measurements were obtained from the database and independently reviewed by two surgeons (CKL and BJM). Discrepancies were resolved through consensus discussion.

### 2.2. Assessment of Clinical Outcomes

Clinical outcomes were evaluated using a patient-reported visual analog scale for neck pain (VAS) and the Japanese Orthopedic Association (JOA) score. Data were assessed preoperatively and at least 24 months postoperatively. The JOA recovery ratio was calculated as: JOA recovery ratio (%) = (postoperative JOA score − preoperative JOA score)/[17 (total score) − preoperative JOA score].

### 2.3. Surgical Procedures

Surgical procedures were determined based on neurological severity, radiological findings, patient comorbidities, and the surgeon’s clinical judgment [5]. Anterior decompression was generally considered when ventral compression due to OPLL was present. When a high COR (≥50%) was observed, anterior decompression was considered after a thorough evaluation of the patient’s comorbidities. For more than three levels of OPLL, LP was preferred in patients with preserved preoperative lordosis, whereas LF was performed in patients with kyphotic alignment greater than 10° to minimize further loss of lordosis.

Anterior decompression involved multilevel anterior cervical discectomy and fusion (ACDF) or anterior cervical corpectomy and fusion (ACCF). In ACDF, the disc and ossified ligament were removed, and the margins were shaped in a wedge. In ACCF, the disc and affected vertebral bodies were removed, and the OPLL was thinned and removed when possible. If complete removal was not feasible, the OPLL was floated anteriorly away from the dural sac.

LP was performed through a midline approach with bilateral muscle detachment, removal of the spinous process, creation of unilateral gutters, and fixation using posterior cervical titanium miniplates (Centerpiece, Medtronic Sofamor Danek, Memphis, TN, USA) with the “door” maintained in the open position.

LF involved laminectomy with posterior fusion using lateral mass screws (C1 and C3–6) and pedicle screws (C2 and C7) with autologous bone grafting. When OPLL extended to C7 or T1, posterior instrumentation was extended across the cervicothoracic junction. Postoperatively, all patients wore either a Miami collar or a Philadelphia brace for approximately three months. Follow-up included clinical and radiographic evaluations 1, 3, and 6 months postoperatively and every 6 months thereafter.

Because this multicenter cohort includes inherent heterogeneity in surgical indications and procedural variations (e.g., ACDF vs. LP vs. LF), all analyses were adjusted for baseline demographic and radiologic differences. Post hoc multiple comparisons were conducted after ANOVA to identify specific pairwise differences, and complication rates were reported as proportions. The heterogeneity was further addressed through multivariate regression to control for confounding variable factors.

### 2.4. Statistical Analysis

Continuous variables are presented as the mean ± standard deviation (SD), and categorical variables are presented as counts and percentages. The normality of data distribution was assessed using the Shapiro–Wilk test. Normally distributed continuous variables were compared using Student’s *t*-test or one-way analysis of variance (ANOVA), whereas non-normally distributed data were analyzed using the Mann–Whitney U test or Kruskal–Wallis test, as appropriate. When ANOVA or Kruskal–Wallis testing indicated significant overall differences among the three surgical groups, Student–Newman–Keuls post hoc tests were performed to identify specific pairwise differences. Categorical variables were compared using the chi-square test or Fisher’s exact test when the expected frequencies were <5. To explore factors associated with neurological recovery, multivariate linear regression analyses were performed using a stepwise selection method. Variables with significant or clinically relevant associations in univariate analyses were entered into the models to adjust for potential confounding related to baseline demographic, radiologic, and procedural differences. Multicollinearity among radiographic variables was assessed using variance inflation factors (VIFs), and variables with VIF > 10 were excluded from the final models. For radiological measurements, interobserver reliability was assessed using Cohen’s κ coefficient for categorical variables and intraclass correlation coefficients (ICCs) for continuous parameters, calculated using a two-way mixed-effects model with consistency agreement and 95% confidence intervals. Agreement levels were categorized as follows: poor (0.00–0.20), fair (0.21–0.40), moderate (0.41–0.60), good (0.61–0.80), and very good (0.81–1.00) [11]. To further mitigate confounding by indication arising from non-randomized surgical selection among surgical treatment groups, a propensity score-based inverse probability of treatment weighting (IPTW) analysis was performed. Propensity scores for the three surgical strategies were estimated using a multinomial logistic regression model incorporating baseline demographic and preoperative radiologic variables. Stabilized IPTW weights were applied to create a weighted pseudo-population in which baseline covariates were balanced across the three groups. To reduce the influence of extreme weights, truncation at the 1st and 99th percentiles was implemented. Weighted comparisons of clinical outcomes, including JOA scores and pain-related measures, were conducted using weighted linear regression models with robust standard errors. This multinomial IPTW approach allowed simultaneous adjustment across all three surgical groups and provided an association-based comparison of outcomes while acknowledging that residual confounding inherent to the retrospective design could not be fully eliminated. All tests were two-sided, and *p*-values < 0.05 were considered statistically significant. All statistical analyses were performed using MedCalc version 23.2.8 (MedCalc Software Ltd., Ostend, Belgium).

## 3. Results

A total of 535 patients (153 males and 382 females) underwent surgical treatment for multilevel cervical OPLL: ACDF (n = 183), LP (n = 238), or LF (n = 114). Of the 367 patients classified as NK-line (+), 181 (49.3%) were FK-line (−) on flexion radiographs. Ultimately, 186 patients were in the NK-line (+)/FK-line (+) (Group 1), 181 in the NK-line (+)/FK-line (−) (Group 2), and 168 in the NK-line (−)/FK-line (−) (Group 3). The mean follow-up duration was 43.2 ± 18.7 months (range, 25–64 months), and the mean age at surgery was 57.32 ± 10.17 years (range, 34–82 years). Baseline demographic and clinical characteristics stratified by dynamic K-line status are summarized in Supplementary Table S1.

### 3.1. Baseline Clinical and Radiological Characteristics According to Dynamic K-Line Status

Group 1 demonstrated the highest JOA recovery rate, compared with Groups 2 and 3 (65.09% vs. 58.0% vs. 54.12%; *p* = 0.0053) (Supplementary Table S1). COR was significantly lower in Group 1 than in Groups 2 and 3 (44.89% vs. 46.24% vs. 51.04%; *p* < 0.001), indicating more severe anterior spinal cord compression in patients with persistent K-line negativity. Group 3 exhibited a smaller C2–7 CA and a greater C2S than the other groups, reflecting more pronounced kyphotic alignment (Supplementary Table S1). The FKD was greatest in Group 1, compared with Groups 2 and 3 (1.38 mm vs. 0.81 mm vs. 0.07 mm; *p* < 0.001), suggesting decreased capacity for spinal cord shift in patients with dynamic K-line negativity.

### 3.2. Clinical and Radiological Outcomes by Surgical Procedure in Patients with Neutral K-Line (+) and Flexion K-Line (+)

In patients with maintained K-line positivity (Group 1), ACDF achieved a significantly higher JOA recovery ratio than LP and LF (77.21% vs. 57.92% vs. 60.77%; *p* = 0.0023) (Table 1). ACDF preserved cervical alignment most effectively. All procedures reduced ROM postoperatively, with the greatest restriction observed in the LF group. Overall, ACDF provided the most favorable neurological recovery and sagittal alignment, LP best preserved cervical ROM, and LF resulted in the greatest reduction in motion.

### 3.3. Clinical and Radiological Outcomes by Surgical Procedure in Patients with Neutral K-Line (+) and Flexion K-Line (−)

**Figure 4:**
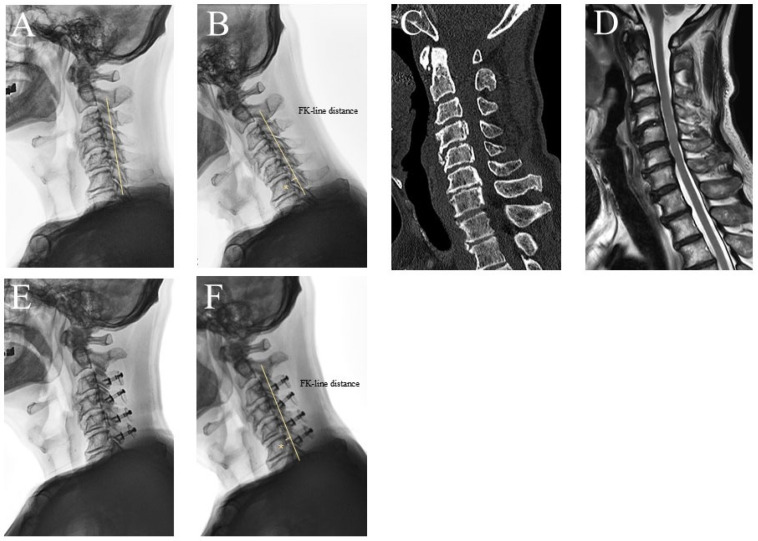
Representative case of LP in a patient with NK-line (+) and FK-line (−). A 68-year-old man with cervical OPLL involving C3–7 presented with bilateral forearm numbness, weakness, and gait disturbance. (**A**) Preoperative neutral lateral radiograph shows preserved lordotic alignment with a positive neutral K-line (NK-line [+]). (**B**) Flexion radiograph reveals a negative flexion K-line (FK-line [−]) with an FK-line distance of 5.2 mm (asterisk). (**C**) Sagittal CT demonstrates segmental-type OPLL extending from C3 to C7. (**D**) T2-weighted sagittal MRI reveals severe ventral cord compression. (**E**) Postoperative neutral radiograph following LP (C3–C6) demonstrates loss of cervical lordosis. (**F**) Postoperative flexion radiograph shows a slightly reduced FK-line distance (4.3 mm; asterisk), consistent with limited posterior cord shift. The JOA score improved from 12 to 14 at the 2-year follow-up (recovery rate: 40%).

In patients with latent dynamic cord compression (Group 2), ACDF resulted in a significantly higher JOA recovery ratio than LP and LF (65.39% vs. 46.43% vs. 62.32%; *p* < 0.001) (Table 2). Preoperative JOA scores were lowest in the LF group, and postoperative gains were greatest with LF, compared with ACDF and LP. ACDF produced the greatest improvement in C2–7 CA, and both LP and LF were associated with a loss of lordosis. ACDF also showed the largest increase in postoperative ΔFKD (Δ 5.96 mm), followed by LF (Δ 5.69 mm), with LP showing minimal correction (Δ 1.84 mm; *p* < 0.001) (Figure 4). ACDF was associated with the greatest improvement in neurological recovery and sagittal alignment, whereas LF showed the highest JOA gain, but at the expense of reduced motion.

### 3.4. Clinical and Radiological Outcomes by Surgical Procedure in Patients with Neutral and Flexion K-Line (−)

In patients with fixed K-line (−) status (Group 3), ACDF demonstrated significantly better neurological outcomes than LP and LF (71.66% vs. 43.41% vs. 56.42%; *p* < 0.001) (Table 3). Both ACDF and LF showed substantial increases in ΔFKD (Δ4.60 mm vs. Δ5.15 mm), whereas LP resulted in minimal improvement (Δ0.81 mm; *p* < 0.001). ACDF achieved the greatest correction of cervical kyphosis. LF showed better recovery than LP but required more extensive surgery. Representative cases are illustrated in Supplementary Figure S2.

### 3.5. Predictors of Neurological Recovery Based on K-Line Status

Stepwise multivariate regression analysis identified distinct factors associated with neurological recovery across the three dynamic K-line groups (Table 4). Multicollinearity among included variables was assessed using variance inflation factors (VIFs). In Group 1, younger age and fewer operative levels showed significant predictive associations with neurological recovery. In Group 2, a larger preoperative C2–7 CA, greater preoperative FKD, and greater postoperative ΔFKD were positively associated with recovery, whereas a greater preoperative flexion CA showed a negative association. In Group 3, both FKD and ΔFKD demonstrated strong positive associations with neurological recovery, while a high COR and a large ΔC2–7 CA were associated with poorer outcomes. Surgical procedure also remained significantly associated with neurological recovery in this group, suggesting that procedural selection is an important factor in patients with persistent K-line (−).

The remaining radiological outcomes based on the surgical procedure and dynamic K-line status are presented (Supplementary Table S2). The incidence of postoperative C5 palsy differed significantly across surgical procedures, with the highest rates observed after LF (Supplementary Table S3).

### 3.6. Interobserver Reliability

Interobserver agreement for radiological measurements showed consistently high reliability across all evaluated parameters. The intraclass correlation coefficients (ICCs) ranged from 0.85 to 0.97, demonstrating good to excellent reliability for cervical sagittal alignment metrics, including C2–7 CA, C2–7 SVA, C2S, and T1S. Both preoperative and postoperative cervical ROM showed excellent reproducibility (ICC = 0.94–0.95), and the COR exhibited near-perfect agreement (ICC = 0.97). All 95% confidence intervals were narrow, further supporting measurement consistency between the two independent observers. These results confirm that the radiographic variables used in the analysis were measured with high methodological rigor and minimal observer-related variability (Supplementary Table S4).

### 3.7. IPTW-Weighted Clinical and Radiological Outcomes According to Surgical Technique

To mitigate confounding by indication arising from non-randomized surgical selection among ACDF, LP, and LF, a multinomial propensity score-based inverse probability of treatment weighting (IPTW) analysis was performed using baseline demographic and preoperative radiologic variables. After weighting, covariate balance across the three surgical groups was substantially improved.

In the IPTW-weighted analysis, all three surgical techniques were associated with significant postoperative improvement in neurological function and neck pain. Compared with LP, ACDF was associated with higher adjusted neurological recovery across dynamic K-line subgroups, whereas LF demonstrated significantly greater neurological recovery than LP in patients with latent or fixed FK-line negativity. Differences in neck pain improvement between the two posterior approaches were modest after weighting.

With respect to radiological outcomes, ACDF and LF were associated with greater improvement in flexion-related parameters, including FK-line distance, compared with LP. In contrast, LP was associated with better preservation of cervical motion in the weighted analysis. Detailed IPTW-weighted clinical and radiological outcomes are presented in Supplementary Table S5.

## 4. Discussion

We investigated the clinical and radiological significance of surgical procedures in patients with multilevel cervical OPLL stratified by dynamic K-line status. Progressive K-line negativity was associated with greater sagittal imbalance, increased anterior cord compression, and diminished neurological recovery. Within this framework, ACDF was associated with more favorable neurological outcomes, while, among patients with latent or fixed FK-line (−), LF demonstrated better recovery than LP. Both preoperative FKD and postoperative FKD change showed significant predictive associations with neurological recovery. However, postoperative FKD change should be interpreted as an imaging marker reflecting the adequacy of decompression rather than as a preoperative determinant of outcome. Although the cohort includes various anterior and posterior procedures, this diversity reflects current surgical practices in multilevel OPLL. By adjusting for preoperative imbalance and conducting subgroup analyses based on dynamic K-line status, we were able to identify meaningful clinical patterns despite procedural variation.

Fujiyoshi et al. introduced the K-line, reporting that patients with K-line (+) OPLL had a 66% recovery rate, whereas those with K-line (−) OPLL achieved only 13.9%, which they attributed to insufficient posterior spinal cord shift after LP [4]. Subsequent investigations demonstrated that preoperative K-line (−) is associated with poor neurological recovery, kyphotic progression, and a high COR [12]. However, conventional K-line assessment is static and does not reflect dynamic cervical motion.

Few studies have investigated fluctuations in the K-line during flexion or extension [6,7]. Even among patients who are K-line (+) in a neutral position, K-line (−) during neck flexion has been associated with poor outcomes following posterior decompression [6,7]. In contrast, Li et al. found that LP was safe and effective in a small group of patients with neutral K-line (−) and K-line (+) during neck extension [13]. During neck flexion, the spinal cord shifts anteriorly and elongates, reducing the anteroposterior diameter, as observed in histologic sections [14]. This results in increased ventral compression and mechanical stress against osteophytes, particularly in patients with sagittal imbalance or kyphotic alignment [14,15]. High cervical kyphosis during flexion has been shown to predict postoperative kyphotic deformity and significant loss of cervical lordosis [16]. The FK-line might better predict clinical and radiological outcomes by reflecting preoperative kyphosis under neck flexion stress, thereby providing a more accurate prognostic indicator than the NK-line.

Han et al. reported no difference in the recovery rate between K-line flexion and extension statuses following ACDF, although patients with extension K-line (−) exhibited high COR [17]. However, their analysis did not stratify outcomes by surgical procedure or identify predictors within each dynamic K-line subgroup. To the best of our knowledge, this is the first study to comprehensively stratify surgical outcomes among ACDF, LP, and LF based on dynamic K-line status and to identify distinct predictors of neurological recovery within each subgroup.

Previous studies have reported comparable outcomes between ACDF and LP in K-line (+) OPLL, with LP offering better motion preservation and lower complication and reoperation rates [17,18]. ACDF, on the other hand, has demonstrated a higher recovery rate and shorter hospital stays than LF due to reduced operative time, blood loss, and muscle dissection [19,20]. In this study, patients with maintained K-line (+) in both the neutral and flexion positions exhibited preserved potential for spinal cord shift, with LP most effectively preserving motion, and LF resulting in the greatest loss of mobility. Our multivariate analysis confirmed that younger age and fewer operative levels independently predicted better recovery in this group, consistent with previous reports [21]. These findings suggest that, in patients with consistent K-line (+) status, patient age and the number of operative levels might outweigh the influence of COR, alignment parameters, or surgical approach.

Posterior decompression is generally effective in patients who maintain K-line (+) status [3,19]. However, some patients with preoperative neutral K-line (+) still experience suboptimal neurological recovery after LP [7,22,23]. Specifically, patients who are K-line (+) in the neutral position but K-line (−) during flexion (FK-line [−]) exhibited worse outcomes after LP than those with consistent K-line (+) status [6,7,24]. Sakai et al. demonstrated that even patients with neutral K-line (+) can have poor outcomes, particularly in the presence of preoperative kyphosis or high K-line tilt [25]. They noted that LP is not suitable when the K-line tilt exceeds 20°, even in K-line (+) OPLL patients [24]. In our study, 49.32% of patients with neutral K-line (+) changed to K-line (−) during flexion. In patients with latent dynamic cord compression, ACDF resulted in the highest recovery rate, possibly by directly alleviating ventral compression and allowing a posterior cord shift. Although LF showed the greatest improvement in JOA scores, it was associated with a greater restriction of cervical motion than LP.

Koda et al. found that postoperative conversion of K-line status from (−) to (+) was associated with improved outcomes from LF in patients with K-line (−) OPLL [26,27]. Similarly, Kim et al. reported that conversion to K-line (+) after surgery correlated with better neurological outcomes in K-line (−) OPLL patients [2]. In our study, ACDF and LF demonstrated greater postoperative increases in FKD than LP, suggesting that LP might be insufficient to address latent anterior cord compression. In contrast, ACDF and LF can provide more reliable decompression, consistent with previous findings [2,20,26]. An increase in FKD after surgery reflects the conversion of K-line status from (−) to (+) because it signifies improved spatial clearance between the OPLL mass and the FK-line. These findings might explain the superior neurological recovery observed with LF, compared with LP, in FK-line (−) patients. In our multivariate analysis, larger FKD and greater postoperative change—indicators of dynamic decompression capacity—were positively associated with neurological recovery in this group, whereas greater flexion C2–7 CA was negatively correlated. These results suggest that favorable baseline sagittal alignment and dynamic decompression capacity, as reflected by both FKD and its postoperative change, support neurological improvement, whereas excessive flexion deformity can impair recovery potential.

In patients with persistent K-line (−) status in both the neutral and flexed positions, the OPLL mass crosses the K-line regardless of cervical movement. These patients, especially those with preoperative kyphosis, are at high risk of postoperative loss of cervical lordosis, progressive kyphotic deformity, and suboptimal outcomes due to limited posterior cord shift following LP [28,29]. Inadequate spinal cord shift can result in residual ventral compression and the exacerbation of neurological deficits [30]. ACDF is generally recommended for cases with significant kyphosis because it enables direct decompression, restoration of lordosis, and improved neurological recovery, compared with posterior approaches [31]. Previous studies have reported that the average JOA score recovery rate after LF in K-line (−) OPLL is approximately 40%, which is superior to LP but inferior to ACDF [32].

In our study, ACDF achieved the greatest correction of cervical kyphosis, and LF produced better recovery than LP, albeit with more extensive procedures. These findings align with previous reports [1,5,32]. Importantly, higher COR and greater kyphotic progression after surgery (ΔC2–7 CA) were negatively associated with recovery, and greater FKD and its postoperative increase correlated positively with neurological improvement. Notably, the surgical modality remained an independent factor, underscoring the critical role of appropriate approach selection in patients with persistent K-line (−) OPLL.

Based on our results, both the preoperative FKD and its postoperative increase were associated with neurological recovery in patients with latent or fixed FK-line (−) status. Miyazaki et al. reported that a larger preoperative difference in K-line distance between extension and flexion—reflecting excessive dynamic displacement—was associated with poorer recovery following LP. However, their study did not assess postoperative changes or include other surgical procedures [33]. In contrast, our findings demonstrate that the postoperative increase in FKD reflects the extent of surgical decompression and the capacity for posterior cord shift. A larger preoperative FKD and its further increase after surgery were both associated with improved neurological outcomes, highlighting the importance of the residual space for cord shift when determining surgical efficacy.

Although FKD may be influenced by patient tolerance and the degree of cervical motion achieved during dynamic radiography, measurements in this study were obtained only in patients who could safely tolerate neck motion without neurological deterioration. Accordingly, FKD should be regarded as a tool for preoperative risk stratification and prognostic assessment, rather than as a sole determinant of surgical approach selection.

Postoperative C5 palsy is a recognized complication attributed to spinal cord shifting and subsequent traction on the cord or nerve roots. The reported incidence ranges from 0% to 13.6% [34,35]. Aggressive kyphosis correction or excessive posterior cord shift after ACDF or LF can exacerbate this risk, particularly when the postoperative ΔFKD is large. Previous studies have associated C5 palsy with the “tethering phenomenon,” reporting significantly greater cord shift at C5 in patients with palsy (5.1 mm) than in those without (2.4 mm) [36]. However, Pennington et al. found that the posterior cord shift was not an independent predictor of postoperative C5 palsy [37]. In our study, ACDF and LF were associated with greater posterior cord shift (3.92 mm and 4.85 mm, respectively) than LP (0.91 mm). The incidence of C5 palsy differed significantly among the surgical groups. These findings align with previous reports [14,36], suggesting that although increased cord shift might contribute to C5 palsy, most cases are transient and resolve with rehabilitation.

Our findings support the clinical utility of dynamic K-line classification as a prognostic tool that provides additional value beyond static assessment. In FK-line (−) patients, both preoperative and postoperative FKD were associated with neurological recovery. Postoperative changes in FKD reflect the adequacy of decompression rather than preoperative independent determinants of neurological recovery. Accordingly, the associations identified in multivariable regression should be interpreted as predictive relationships inherent to a retrospective study, rather than as evidence of direct causal effects.

Importantly, although propensity score-based IPTW analysis was performed to mitigate confounding by indication, the present findings should be interpreted as association-based rather than causal effects of specific surgical procedures. Surgical selection in multilevel cervical OPLL is inherently influenced by disease severity, anatomical constraints, and surgeon judgment, which cannot be fully eliminated in a retrospective design. Dynamic K-line stratification should be regarded as a complementary decision-support and risk-assessment tool that informs surgical planning, rather than a definitive determinant of surgical approach selection.

**Figure 5:**
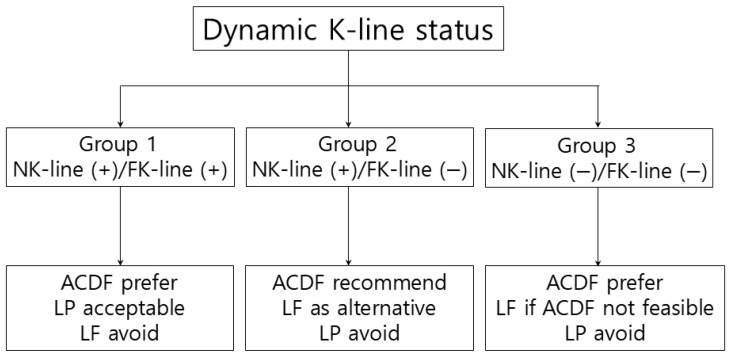
Treatment algorithm based on dynamic K-line status in multilevel cervical OPLL. Surgical selection should be guided by dynamic cord compression patterns and sagittal alignment. Anterior decompression is generally favored across all K-line groups due to superior neurological recovery. LP is acceptable in patients with maintained K-line (+) and preserved cervical lordosis. LF is recommended in latent or fixed FK-line (−) when anterior decompression is contraindicated or not feasible.

Although ACDF was associated with more favorable neurological outcomes, LF offered a favorable balance between surgical invasiveness and neurological benefit, particularly in patients with latent or fixed FK-line (−) status. On this basis, we propose a surgical decision-making algorithm based on dynamic K-line status to support individualized treatment selection in multilevel cervical OPLL (Figure 5).

This study has several limitations. First, its retrospective design carries the inherent risk of selection bias, particularly given differences in surgical indications for anterior decompression, LP, and LF. Second, the heterogeneity of surgical techniques represents a methodological limitation; however, such variability is common in multicenter OPLL cohorts because of the rarity and complexity of the disease. To address this issue, we performed a multinomial inverse probability of treatment weighting (IPTW) analysis to adjust simultaneously for all three surgical groups. This approach provided an association-based comparison of outcomes, reduced baseline imbalances, and enhanced the validity of the comparative analysis. Third, radiographic assessments were limited to standard static and flexion-extension radiographs, without MRI-based confirmation of cord shift. Integrating MRI measurements, such as cord cross-sectional area, with dynamic K-line metrics could further refine prognostication. Fourth, although the mean follow-up period was 43.2 months, longer-term studies are required to evaluate the durability of neurological recovery and radiographic progression. Prospective, longitudinal studies with standardized imaging protocols are warranted to validate our findings and refine surgical selection criteria based on dynamic K-line status.

## 5. Conclusions

Dynamic K-line classification provides prognostic value superior to static assessment in multilevel cervical OPLL. Progressive K-line negativity was associated with increased sagittal imbalance, greater anterior cord compression, and diminished neurological recovery. ACDF showed consistently favorable associations with favorable neurological improvement and sagittal alignment correction across dynamic K-line subgroups. In patients with latent or fixed FK-line (−), LF was associated with better neurological recovery than LP, despite reduced postoperative mobility. Notably, preoperative FK-line distance showed a significant association with neurological improvement, and an increase in FK-line distance after surgery reflected the adequacy of decompression. These findings support the clinical integration of dynamic K-line metrics into surgical planning as a prognostic and decision-support tool to optimize treatment strategies and improve outcomes.

## Figures and Tables

**Table 1:** Clinical outcomes and cervical alignment changes by surgical procedure in patients with NK-line (+) and FK-line (+).

A. Clinical Outcomes by Surgical Procedure				
**Variable**	ACDF (n = 65)	LP (n = 93)	LF (n = 28)	***p* Value**
**JOA score**				
Preop	11.89 ± 2.59	12.51 ± 3.16	13.11 ± 1.73	0.023 *
Final	15.82 ± 1.42	14.68 ± 3.27	15.46 ± 1.11	0.068
Change	3.92 ± 2.29	2.17 ± 2.44	2.36 ± 1.37	<0.001 *
**Recovery ratio (%)**	77.21 ± 25.14	57.92 ± 39.52	60.77 ± 27.11	0.002 *
**Neck VAS**				
Preop	4.75 ± 2.33	3.11 ± 2.59	4.18 ± 2.64	0.002 *
Final	1.63 ± 1.23	2.45 ± 2.27	2.96 ± 1.71	0.004 *
Change	3.12 ± 2.07	0.66 ± 2.88	1.21 ± 2.01	<0.001 *
**No. of operative levels**	2.28 ± 0.48	3.46 ± 0.94	3.79 ± 0.96	<0.001 *
**B. Radiological outcomes by surgical procedure**				
**Variable**	ACDF (n = 65)	LP (n = 93)	LF (n = 28)	***p* Value**
**COR** (%)	44.35 ± 11.08	44.98 ± 14.39	45.83 ± 13.18	0.879
**FKD** (mm)				
Preop	1.41 ± 2.25	1.06 ± 1.64	3.03 ± 1.10	0.001 *
Final	2.51 ± 3.90	1.54 ± 2.72	5.35 ± 2.25	<0.001 *
Change	1.09 ± 2.12	0.48 ± 1.72	2.31 ± 1.47	<0.001 *
**C2–7 CA** (°)				
Preop	14.25 ± 9.18	12.01 ± 9.26	14.36 ± 5.98	0.218
Final	14.99 ± 8.92	7.69 ± 10.15	7.64 ± 8.66	<0.001 *
Change	−0.43 ± 8.39	−4.64 ± 7.43	−6.72 ± 10.49	0.001 *
**C2–7 flex CA** (°)				
Preop	−12.19 ± 10.60	−12.30 ± 10.39	−8.13 ± 8.51	0.067
Final	−2.07 ± 10.16	−6.72 ± 11.34	−3.79 ± 7.99	<0.040 *
Change	9.97 ± 9.31	6.39 ± 10.72	4.35 ± 8.72	0.006 *
**ROM** (°)				
Preop	37.81 ± 12.77	34.02 ± 12.29	31.84 ± 12.33	0.059
Final	24.36 ± 9.78	21.50 ± 12.22	15.13 ± 8.53	0.002 *
Change	−15.19 ± 14.81	−15.31 ± 15.82	−16.70 ± 10.49	0.900

ACDF = anterior cervical discectomy and fusion; CA = Cobb angle; C2–7 flex CA = C2–7 flexion Cobb angle; COR = canal occupying ratio; FKD = FK-line distance, i.e., the distance from the flexion K-line to the posterior vertebral body line; FK-line = flexion K-line; JOA = Japanese Orthopedic Association; LF = laminectomy with fusion; LP = laminoplasty; NK-line = neutral K-line; ROM = range of motion; VAS = visual analog scale. All data are expressed as the mean ± SD unless otherwise noted. * *p* < 0.05.

**Table 2:** Clinical outcomes and cervical alignment changes by surgical procedure in patients with NK-line (+) and FK-line (−).

**A. Clinical outcomes by surgical procedure**				
**Variable**	ACDF (n = 73)	LP (n = 63)	LF (n = 45)	***p* Value**
**JOA score**				
Preop	13.31 ± 1.78	12.78 ± 1.94	11.91 ± 2.47	0.002 *
Final	15.79 ± 1.58	14.68 ± 1.93	15.02 ± 1.53	<0.001 *
Change	2.49 ± 1.86	1.90 ± 1.71	3.11 ± 1.87	0.004 *
**Recovery ratio (%)**	65.39 ± 46.28	46.43 ± 40.65	62.32 ± 24.85	<0.001 *
**Neck VAS**				
Preop	5.72 ± 2.52	3.48 ± 2.55	4.73 ± 2.67	<0.001 *
Final	2.65 ± 1.35	2.30 ± 1.96	2.55 ± 1.68	0.152
Change	3.07 ± 2.43	1.18 ± 2.64	2.18 ± 2.55	<0.001 *
**No. of operative levels**	2.08 ± 0.46	3.62 ± 0.92	3.98 ± 0.93	<0.001 *
**B. Radiological outcomes by surgical procedure**				
**Variable**	ACDF (n = 73)	LP (n = 63)	LF (n = 45)	***p* Value**
**COR** (%)	47.04 ± 13.44	46.14 ± 14.33	45.03 ± 11.33	0.730
**FKD** (mm)				
Preop	1.19 ± 2.53	0.29 ± 1.56	0.51 ± 2.91	0.131
Final	7.15 ± 3.09	2.31 ± 3.39	6.21 ± 2.64	<0.001 *
Change	5.96 ± 3.41	1.84 ± 3.31	5.69 ± 2.16	<0.001 *
**C2–7 CA** (°)				
Preop	6.14 ± 9.38	8.08 ± 8.43	12.88 ± 7.40	<0.001 *
Final	9.87 ± 6.65	4.99 ± 8.61	7.02 ± 7.01	<0.001 *
Change	0.13 ± 10.79	−3.08 ± 6.93	−5.86 ± 6.79	<0.001 *
**C2–7 flex CA** (°)				
Preop	−21.43 ± 10.03	−18.38 ± 10.65	−12.51 ± 8.83	<0.001 *
Final	−11.78 ± 8.52	−11.69 ± 12.17	−5.03 ± 5.77	<0.006 *
Change	14.01 ± 9.09	7.66 ± 10.83	7.42 ± 8.78	0.002 *
**ROM** (°)				
Preop	42.68 ± 11.99	35.90 ± 14.04	36.29 ± 11.52	0.006 *
Final	32.91 ± 11.57	23.24 ± 12.29	17.71 ± 10.30	<0.001 *
Change	−21.94 ± 16.73	−15.01 ± 14.75	−18.16 ± 11.75	0.049 *

ACDF = anterior cervical discectomy and fusion; CA = Cobb angle; C2–7 flex CA = C2–7 flexion Cobb angle; COR = canal occupying ratio; FKD = FK-line distance, i.e., the distance from the flexion K-line to the posterior vertebral body line; FK-line = flexion K-line; JOA = Japanese Orthopedic Association; LF = laminectomy with fusion; LP = laminoplasty; NK-line = neutral K-line; ROM = range of motion; VAS = visual analog scale. All data are expressed as the mean ± SD unless otherwise noted. * *p* < 0.05.

**Table 3:** Clinical outcomes and cervical alignment changes by surgical procedure in patients with NK-line (−) and FK-line (−).

**A. Clinical outcomes by surgical procedure**				
**Variable**	ACDF (n = 45)	LP (n = 82)	LF (n = 41)	***p* Value**
**JOA score**				
Preop	12.62 ± 2.31	13.15 ± 1.82	11.31 ± 2.56	0.002 *
Final	15.84 ± 1.19	14.89 ± 1.62	14.36 ± 2.46	<0.001 *
Change	3.22 ± 2.11	1.77 ± 1.51	3.05 ± 1.99	<0.001 *
**Recovery ratio (%)**	71.66 ± 28.51	43.41 ± 35.63	56.42 ± 33.36	<0.001 *
**Neck VAS**				
Preop	5.02 ± 2.41	4.16 ± 2.73	4.28 ± 2.98	0.204
Final	2.47 ± 1.56	2.39 ± 1.69	2.46 ± 1.76	0.971
Change	2.55 ± 1.87	1.77 ± 2.45	1.82 ± 2.65	0.102
**No. of operative levels**	2.38 ± 0.57	3.35 ± 0.78	4.59 ± 1.21	<0.001 *
**B. Radiological outcomes by surgical procedure**				
**Variable**	ACDF (n = 45)	LP (n = 82)	LF (n = 41)	***p* Value**
**COR** (%)	55.18 ± 13.64	49.59 ± 14.22	49.24 ± 14.00	0.136
**FKD** (mm)				
Preop	0.25 ± 3.95	0.22 ± 1.42	0.20 ± 3.84	0.103
Final	4.65 ± 4.50	1.15 ± 2.61	5.35 ± 3.35	<0.001 *
Change	4.60 ± 5.38	0.81 ± 1.93	5.15 ± 2.74	<0.001 *
**C2–7 CA** (°)				
Preop	3.74 ± 9.41	8.47 ± 11.29	6.03 ± 8.89	0.022 *
Final	6.93 ± 5.83	6.11 ± 11.24	3.10 ± 8.27	0.117
Change	1.28 ± 8.02	−2.30 ± 8.64	−2.93 ± 8.55	0.034 *
**C2–7 flex CA** (°)				
Preop	−21.02 ± 10.65	−16.89 ± 12.10	−13.53 ± 10.44	0.012 *
Final	−8.37 ± 6.91	−10.42 ± 12.77	−7.90 ± 8.12	0.269
Change	14.82 ± 12.26	6.99 ± 11.01	5.63 ± 10.13	<0.001 *
**ROM** (°)				
Preop	36.83 ± 13.69	35.89 ± 13.36	27.86 ± 11.68	0.004 *
Final	22.58 ± 10.49	22.59 ± 13.48	14.59 ± 8.75	0.001 *
Change	−21.10 ± 16.61	−14.48 ± 16.11	−13.27 ± 12.44	0.038 *

ACDF = anterior cervical discectomy and fusion; CA = Cobb angle; C2–7 flex CA = C2–7 flexion Cobb angle; COR = canal occupying ratio; FKD = FK-line distance, i.e., the distance from the flexion K-line to the posterior vertebral body line; FK-line = flexion K-line; JOA = Japanese Orthopedic Association; LF = laminectomy with fusion; LP = laminoplasty; NK-line = neutral K-line; ROM = range of motion; VAS = visual analog scale. All data are expressed as the mean ± SD unless otherwise noted. * *p* < 0.05.

**Table 4:** Multivariate regression analysis of neurological outcomes, patient characteristics, and cervical parameters among groups based on K-line status.

**Group 1: NK-line (+)/FK-line (+)** Model: (R^2^ = 0.327, *p* = 0.004)						
Variables	β-coefficient	SE	*t*-value	*p* value	95% CI	VIF
Age (years)	−0.760	0.369	−2.057	0.042	(−1.494~−0.025)	1.246
No. of operative levels	−19.129	5.142	−3.720	0.004	(−29.355~−8.904)	1.901
**Group 2: NK-line (+)/FK-line (−)** Model: (R^2^ = 0.532, *p* = 0.001)						
Variables	β-coefficient	SE	*t*-value	*p* value	95% CI	VIF
C2–7 flex CA pre	−1.753	0.622	−2.819	0.006	(−2.999~−0.507)	3.336
C2–7 CA pre	1.985	0.708	2.803	0.007	(0.566~3.404)	3.089
FKD pre	5.318	2.058	2.583	0.012	(1.195~9.442)	1.883
ΔFKD	3.678	1.465	2.509	0.015	(0.742~6.614)	1.726
**Group 3: NK-line (−)/FK-line (−)** Model: (R^2^ = 0.385, *p* = 0.002)						
Variables	β-coefficient	SE	*t*-value	*p* value	95% CI	VIF
ΔC2–7 CA	−1.771	0.661	−2.677	0.009	(−3.088~−0.453)	1.085
COR	−1.019	0.333	−3.055	0.003	(−1.683~−0.354)	1.243
FKD pre	7.262	2.122	3.421	0.001	(3.033~11.490)	1.268
ΔFKD	4.270	1.441	2.963	0.004	(1.339~7.141)	1.216
Surgical procedure	−19.644	7.701	−2.551	0.012	(−34.989~−4.299)	1.211

CA = Cobb angle; ΔC2–7 CA = change in C2–7 CA post- and pre-operation; flex CA = C2–7 flexion Cobb angle; COR = canal occupying ratio; FKD = FK-line distance, i.e., the distance from the flexion K-line to the posterior vertebral body line; ΔFKD = change in FKD post- and pre-operation; FK-line = flexion K-line; NK-line = neutral K-line; Pre = preoperative; VIF = variance inflation factor.

## Data Availability

The datasets generated and analyzed during the current study are not publicly available due to privacy concerns and ethical restrictions but are available from the corresponding author upon reasonable request.

## Supplementary Materials

The following supporting information can be downloaded at: https://www.mdpi.com/article/10.3390/jcm15020520/s1, Supplementary Figure S1: Representative case of ACDF in a patient with NK-line (−) and FK-line (−); Supplementary Figure S2: Representative case of LF in a patient with NK-line (−) and FK-line (−); Supplementary Table S1: Baseline clinical and radiological characteristics according to K-line status; Supplementary Table S2: Radiological outcomes by surgical procedure based on dynamic K-line status; Supplementary Table S3: Clinical outcomes and cervical alignment changes by surgical procedures; Supplementary Table S4: Interobserver reliability of radiological parameters; Supplementary Table S5: IPTW-weighted clinical and radiological outcomes according to surgical procedures.

## Abbreviations

The following abbreviations are used in this manuscript:

ACDF	anterior cervical discectomy and fusion
COR	canal-occupying ratio
FKD	FK-line distance
LF	laminectomy with fusion
LP	laminoplasty
OPLL	ossification of the posterior longitudinal ligament
