# Supplementary material for: Dynamic K-Line Status and Surgical Outcomes in Multilevel Cervical OPLL: A Multicenter Comparative Study
Source: J Clin Med. 2026 Jan 8;15(2):520. doi: 10.3390/jcm15020520 (PMC12842165; doi:10.3390/jcm15020520)
Supplement: Supplementary file 1 [file jcm-15-00520-s001.zip › jcm-4045547-supplementary.pdf]

Supplementary Table S1. Baseline clinical and radiological characteristics according to K-line status

	Neutral (+) / Flexion (+) (n=186)	Neutral (+) / Flexion (−) (n=181)	Neutral (−) / Flexion (−) (n=168)	**p value**
**Age** (years)	58.77 ± 9.45	56.78 ± 9.86	57.81 ± 10.49	0.158
**Sex** (M:F)	61:125	43:138	49:119	0.156
**JOA score**				
Pre	12.38 ± 2.81	12.79 ± 2.08	12.57 ± 2.26	0.587
Final	15.19 ± 2.55	15.22 ± 1.76	15.03 ± 1.83	0.039*
Change	2.81 ± 2.39	2.44 ± 1.86	2.46 ± 1.93	0.262
**Recovery ratio** (%)	65.09 ± 34.40	58.00 ± 40.69	54.12 ± 35.19	0.005*
**No. of operative levels**	3.07 ± 1.01	3.08 ± 1.14	3.37 ± 1.16	0.026*
**OPLL type**				0.001*
Segmental	68 (36.56%)	74 (40.88%)	40 (23.81%)	
Continued	44 (23.66%)	36 (19.89%)	47 (27.98%)	
Mixed	58 (31.18%)	44 (24.31%)	69 (41.07%)	
Other	16 (8.60%)	27 (14.92%)	12 (7.14%)	
**COR** (%) pre	44.89 ± 13.08	46.24 ± 13.24	51.04 ± 14.16	<0.001*
**FKD** (mm)	1.38 ± 1.96	0.81 ± 2.39	0.07 ± 2.99	<0.001*
**C2S** (°) pre	12.91 ± 8.67	13.80 ± 7.44	17.33 ± 8.11	<0.001*
**T1S** (°) pre	25.18 ± 7.05	23.93 ± 6.97	23.13 ± 6.63	0.020*
**SVA** (mm) pre	9.16 ± 12.98	18.44 ± 12.69	14.35 ± 14.42	<0.001*
**C2–7 CA** (º) pre	13.14 ± 8.85	8.45 ± 8.96	6.55 ± 10.39	<0.001*
**C2–7 flex CA** (º) pre	-11.63 ± 10.26	-18.72 ± 10.50	-17.36 ± 11.56	<0.001*
**ROM** (º) pre	35.06 ± 12.59	39.21 ± 12.98	34.31 ± 13.49	0.002*

CA = Cobb angle; C2S = C2 slope; C2–7 flex CA = C2–7 flexion Cobb angle; COR = canal occupying ratio; FK-line = flexion K-line; FKD = FK-line distance, i.e., the distance from the flexion K-line to the posterior vertebral body line; JOA = Japanese Orthopedic Association; Pre = preoperative; ROM = range of motion; SVA = sagittal vertical axis; T1S = T1 slope. All data are expressed as the mean ± SD unless otherwise noted. *p < 0.05.

Supplementary Table S2. Radiological outcomes by surgical procedure based on Dynamic K-line status.

**Group 1: NK-line (+)/FK-line (+)**				
**Variable**	ACDF (n=65)	LP (n=93)	LF (n=28)	**p value**
**Operation time** (min)	173.49 ± 70.61	161.72 ± 52.49	192.29 ± 60.99	0.095
**Blood loss** (ml)	525.46 ± 418.65	572.90 ± 659.80	523.96 ± 444.56	0.936
**C2S** (°)				
Preop	11.68 ± 6.94	14.12 ± 9.41	11.89 ± 9.52	0.318
Final	12.65 ± 7.35	17.38 ± 10.50	17.96 ± 11.76	0.006*
Change	1.46 ± 6.96	2.99 ± 8.38	5.16 ± 12.75	0.091
**T1S** (°)				
Preop	24.51 ± 6.71	24.44 ± 7.14	29.13 ± 6.43	0.005*
Final	25.07 ± 7.48	23.86 ± 8.60	28.87 ± 7.08	0.017*
Change	0.91 ± 7.33	-0.84 ± 7.43	-1.35 ± 7.65	0.118
**SVA** (mm)				
Preop	6.76 ± 11.21	8.15 ± 11.84	28.39 ± 13.90	<0.001*
Final	6.25 ± 9.18	9.43 ± 14.25	34.90 ± 15.25	<0.001*
Change	-0.51 ± 4.58	1.21 ± 6.38	6.51 ± 9.04	0.079
**Group 2: NK-line (+)/FK-line (−)**				
**Variable**	ACDF (n=73)	LP (n=63)	LF (n=45)	**p value**
**Operation time** (min)	162.78 ± 84.59	166.56 ± 58.11	191.86 ± 55.82	0.003*
**Blood loss** (ml)	495.20 ± 608.12	430.48 ± 341.20	618.98 ± 468.75	0.009*
**C2S** (°)				
Preop	13.94 ± 7.48	13.12 ± 6.73	14.54 ± 8.39	0.616
Final	13.96 ± 8.55	18.56 ± 9.89	19.87 ± 8.65	<0.001*
Change	-1.99 ± 5.26	5.28 ± 9.01	5.26 ± 10.94	<0.001*
**T1S** (°)				
Preop	21.48 ± 5.80	24.11 ± 7.17	27.89 ± 6.77	<0.001*
Final	23.52 ± 5.98	23.79 ± 8.24	29.36 ± 6.68	<0.001*
Change	2.57 ± 5.01	-0.27 ± 6.78	1.41 ± 7.22	0.041*

**SVA** (mm)				
Preop	19.64 ± 11.68	13.02 ± 13.45	23.53 ± 11.73	<0.001*
Final	21.21 ± 13.21	16.15 ± 15.33	29.25 ± 12.25	<0.001*
Change	1.57 ± 7.80	3.05 ± 8.14	5.72 ± 8.46	0.082

Group 3: NK-line (−)/FK-line (−)

**Variable**	ACDF (n=45)	LP (n=82)	LF (n=41)	**p value**
**Operation time** (min)	191.89 ± 68.55	160.68 ± 49.14	232.82 ± 86.76	<0.001*
**Blood loss** (ml)	613.00 ± 520.44	502.32 ± 384.62	901.03 ± 690.23	<0.001*
**C2S** (°)				
Preop	17.36 ± 6.99	16.34 ± 8.37	19.32 ± 8.65	0.170
Final	15.76 ± 6.19	20.44 ± 9.20	22.30 ± 8.49	0.001*
Change	-0.81 ± 6.43	4.03 ± 9.00	-1.17 ± 10.16	0.010*
**T1S** (°)				
Preop	20.56 ± 6.28	23.62 ± 6.93	25.24 ± 5.49	0.003*
Final	21.51 ± 5.48	22.70 ± 8.19	25.05 ± 6.57	0.071
Change	1.59 ± 4.94	-0.96 ± 6.75	-0.65 ± 9.73	0.198
**SVA** (mm)				
Preop	16.02 ± 14.64	7.14 ± 10.21	26.56 ± 12.54	<0.001*
Final	15.59 ± 14.47	8.89 ± 11.32	32.68 ± 11.67	<0.001*
Change	-0.44 ± 7.56	1.75 ± 5.69	6.12 ± 13.13	0.003*

ACDF = anterior cervical discectomy and fusion; CA = Cobb angle; C2S = C2 slope; C2-7 flex CA = C2-7 flexion Cobb angle; COR = canal occupying ratio; FKD = FK-line distance, i.e., the distance from the flexion K-line to the posterior vertebral body line; Pre = preoperative; ROM = range of motion; RR = Japanese Orthopaedic Association (JOA) recovery ratio; SVA = sagittal vertical axis; T1S = T1 slope. All data are expressed as mean ± SD unless otherwise noted. *P < 0.05.

Supplementary Table S3. Clinical outcomes and cervical alignment changes by surgical procedures

**A. Clinical outcomes by surgical procedure**				
**Variable**	ACDF (n=183)	LP (n=238)	LF (n=114)	**p value**
**JOA score**				
Preop	12.64 ± 2.30	12.80 ± 2.47	11.98 ± 2.42	0.001*
Final	15.81 ± 1.43	14.75 ± 2.46	14.85 ± 19.94	<0.001*
Change	3.17 ± 2.16	1.95 ± 1.97	2.87 ± 1.84	<0.001*
**Recovery ratio (%)**	71.13 ± 35.95	49.88 ± 38.93	59.19 ± 29.24	<0.001*
**Neck VAS**				
Preop	5.20 ± 2.45	3.55 ± 2.64	4.41 ± 2.76	<0.001*
Final	2.25 ± 1.43	2.37 ± 1.99	2.64 ± 1.72	0.121
Change	2.96 ± 2.18	1.18 ± 2.69	1.77 ± 2.68	<0.001*
**No. of operated levels**	2.23 ± 0.51	3.47 ± 0.88	4.18 ± 1.14	<0.001*
**Operation time** (min)	173.68 ± 76.54	162.64 ± 52.76	209.34 ± 77.86	<0.001*
**Blood loss** (ml)	534.70 ± 525.46	510.88 ± 503.25	705.77 ± 579.74	0.004*
**Complications**				
C5 n palsy	5 (2.73%)	8 (3.36%)	10 (8.77%)	0.017*
Hematoma	1 (0.55%)	1 (0.42%)	1 (0.88%)	0.857
Revision	3 (1.64%)	3 (1.26%)	1 (0.88%)	0.593
Dura tear	19 (10.38%)	3 (1.26%)	3 (2.63%)	<0.001*

**B. Radiological outcomes by surgical procedure**				
**Variable**	ACDF (n=183)	LP (n=238)	LF (n=114)	**p value**
**COR** (%)	49.20 ± 13.24	50.61 ± 13.17	52.94 ± 12.63	0.1840
**FKD** (mm)				
Preop	1.38 ± 1.96	0.81 ± 2.39	0.07 ± 2.99	<0.001*
Final	4.93 ± 4.25	1.55 ± 2.85	5.65 ± 2.95	<0.001*
Change	3.92 ± 4.24	0.91 ± 2.29	4.85 ± 2.63	<0.001*
**C2S** (°)				
Preop	14.02 ± 7.46	14.62 ± 8.47	15.56 ± 9.20	0.287
Final	13.96 ± 7.65	18.75 ± 9.95	20.25 ± 9.55	<0.001*

Change	-0.25 ± 6.45	3.92 ± 8.76	3.60 ± 11.46	<0.001*
**T1S** (°)				
Preop	22.31 ± 6.44	24.07 ± 7.05	27.27 ± 6.40	<0.001*
Final	23.55 ± 6.54	23.44 ± 8.35	27.71 ± 6.97	<0.001*
Change	1.63 ± 6.09	-0.75 ± 7.01	0.09 ± 7.99	0.008*
**SVA** (mm)				
Preop	14.23 ± 13.52	8.81 ± 11.77	26.15 ± 12.68	<0.001*
Final	14.56 ± 13.87	10.67 ± 13.68	32.41 ± 13.26	<0.001*
Change	0.34 ± 6.82	1.82 ± 6.56	6.26 ± 11.04	<0.001*
**C2-7 CA** (°)				
Preop	8.34 ± 10.27	9.77 ± 9.92	10.88 ± 8.37	0.018*
Final	11.31 ± 8.19	6.43 ± 10.18	5.92 ± 8.15	<0.001*
Change	0.22 ± 9.31	-3.43 ± 7.77	-4.96 ± 8.54	<0.001*
**C2-7 flex CA** (°)				
Preop	-18.21 ± 11.20	-15.37 ± 11.32	-11.48 ± 9.69	<0.001*
Final	-6.87 ± 9.86	-9.25 ± 12.19	-5.56 ± 7.83	0.003*
Change	12.72 ± 10.22	6.90 ± 10.81	5.89 ± 9.07	<0.001*
**ROM** (°)				
Preop	31.50 ± 12.28	35.12 ± 13.07	39.50 ± 12.91	<0.001*
Final	15.64 ± 9.25	22.31 ± 12.66	26.85 ± 11.40	<0.001*
Change	-15.75 ± 11.71	-14.95 ± 15.60	-19.43 ± 16.27	0.013*

ACDF = anterior cervical discectomy and fusion; CA = Cobb angle; C2S = C2 slope; C2-7 flex CA = C2-7 flexion Cobb angle; COR = canal occupying ratio; FKD = FK-line distance, i.e., the distance from the flexion K-line to the posterior vertebral body line; Pre = preoperative; ROM = range of motion; RR = Japanese Orthopaedic Association (JOA) recovery ratio; SVA = sagittal vertical axis; T1S = T1 slope. All data are expressed as mean ± SD unless otherwise noted. *P < 0.05.

Supplementary Table S4. Interobserver Reliability of Radiological Parameters

Variable	ICC	95% CI
C2-7 CA Preop	0.87	0.82 to 0.89
C2-7 CA Final	0.89	0.86 to 0.92
ROM Preop	0.94	0.92 to 0.96
ROM Final	0.95	0.93 to 0.97
C2-7 SVA Preop	0.94	0.91 to 0.96
C2-7 SVA Final	0.94	0.92 to 0.95
C2S Preop	0.85	0.79 to 0.88
C2S Final	0.97	0.96 to 0.98
T1S Preop	0.96	0.95 to 0.97
T1S Final	0.95	0.94 to 0.96
COR Preop	0.97	0.96 to 0.97

CA = Cobb angle; C2S = C2 slope; CI = confidence interval; COR = canal-occupying ratio; ICC = intraclass confidence correlation; Preop = preoperative; ROM = range of motion; SVA = sagittal vertical axis; T1S = T1 slope.

Supplementary Table S5. IPTW-Weighted Clinical and Radiological Outcomes According to Surgical Procedures

**A. Clinical outcomes by surgical procedure**				
**Variable**	ACDF	LP	LF	**p value**
**JOA score**				
Preop	12.73 ± 2.41	12.92 ± 2.09	12.54 ± 2.53	0.562
Final	15.79 ± 1.50	15.11 ± 1.72	14.89 ± 2.43	<0.001*
Change	3.06 ± 2.22	2.19 ± 1.78	2.35 ± 2.10	0.003*
**Recovery ratio (%)**	69.01 ± 36.70	53.23 ± 42.15	54.80 ± 37.56	0.003*
**Neck VAS**				
Preop	4.55 ± 2.57	4.15 ± 2.68	4.23 ± 3.18	0.501
Final	2.10 ± 1.46	2.72 ± 2.13	2.39 ± 1.87	0.043*
Change	2.45 ± 2.30	1.43 ± 2.50	1.84 ± 3.47	0.006*

**B. Radiological outcomes by surgical procedure**				
**Variable**	ACDF	LP	LF	**p value**
**COR** (%)	47.14 ± 13.69	47.62 ± 14.51	47.83 ± 13.19	0.902
**FKD** (mm)				
Preop	0.92 ± 2.94	0.63 ± 1.64	0.38 ± 2.68	0.308
Final	4.87 ± 4.21	1.76 ± 3.10	3.76 ± 3.34	<0.001*
**C2S** (°)				
Preop	14.05 ± 7.54	13.87 ± 7.80	16.18 ± 9.86	0.068
Final	13.98 ± 7.66	17.84 ± 9.73	21.69 ± 10.48	<0.001*
**T1S** (°)				
Preop	22.47 ± 6.49	23.82 ± 6.72	26.66 ± 7.04	<0.001*
Final	23.48 ± 6.59	23.18 ± 7.37	27.28 ± 9.13	<0.001*
**SVA** (mm)				
Preop	14.58 ± 13.93	9.11 ± 11.72	16.89 ± 13.72	<0.001*
Final	14.90 ± 14.13	11.46 ± 14.34	21.68 ± 16.64	<0.001*
**C2-7 CA** (°)				
Preop	8.34 ± 11.21	10.09 ± 10.87	8.96 ± 12.04	0.516
Final	10.97 ± 10.45	6.72 ± 9.98	2.56 ± 11.02	<0.001*

Change	2.64 ± 6.82	-3.37 ± 7.41	-6.40 ± 8.03	<0.001*
**ROM** (º)				
Preop	38.75 ± 12.98	34.32 ± 12.68	34.11 ± 12.86	<0.001*
Final	26.67 ± 11.34	23.65 ± 12.27	15.28 ± 9.92	<0.001*
Change	-18.87 ± 16.73	-13.67 ± 14.95	-18.78 ± 13.75	0.003*

ACDF = anterior cervical discectomy and fusion; CA = Cobb angle; C2S = C2 slope; C2-7 flex CA = C2-7 flexion Cobb angle; COR = canal occupying ratio; FKD = FK-line distance, i.e., the distance from the flexion K-line to the posterior vertebral body line; IPTW = inverse probability of treatment weighting; LF = laminectomy with fusion; LP = laminoplasty; Pre = preoperative; ROM = range of motion; RR = Japanese Orthopaedic Association (JOA) recovery ratio; SVA = sagittal vertical axis; T1S = T1 slope. All data are expressed as mean ± SD unless otherwise noted. *P < 0.05.

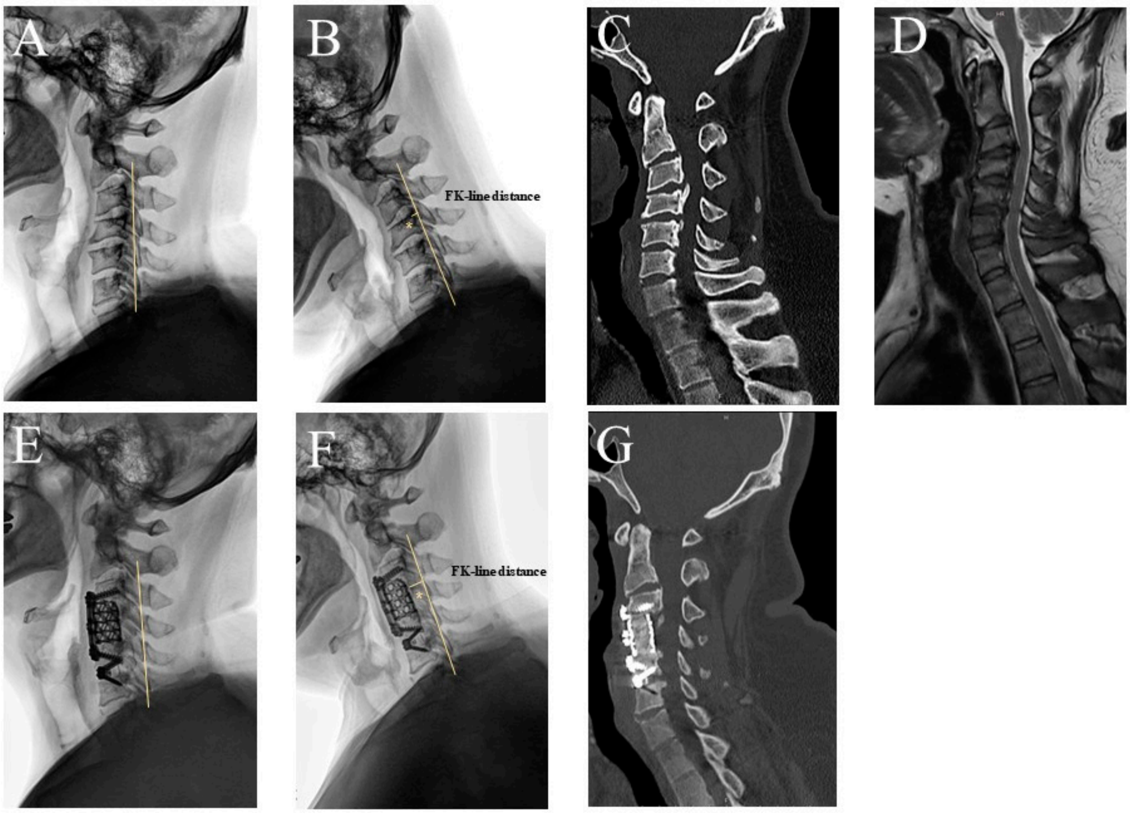

Supplementary Figure S1. Representative case of anterior decompression in a patient with NK-line (−) and FK-line (−).

A 42-year-old man with cervical OPLL from C3 to C6 presented with bilateral arm numbness, weakness, and ataxic gait. (A) Preoperative neutral radiograph shows kyphotic alignment with a negative neutral K-line (NK-line [−]). (B) Flexion radiograph reveals a negative FK-line with a FK-line distance of 1.3 mm (asterisk). (C) Sagittal CT shows mixed-type OPLL at C3–6. (D) T2-weighted sagittal MRI reveals severe ventral cord compression. (E) Postoperative neutral radiograph after C4 corpectomy and C5–C6 segmental fusion shows correction of cervical kyphosis and conversion of the K-line from (−) to (+). (F) Flexion radiograph demonstrates increased FK-line distance (12.1 mm; asterisk). (G) Postoperative CT confirms adequate decompression and canal widening. The JOA score improved from 13 to 17 at 2-year follow-up (recovery rate: 100%). CT = computed tomography; FK-line = flexion K-line; LP = laminoplasty; MRI = magnetic resonance imaging; OPLL = ossification of the posterior longitudinal ligament.

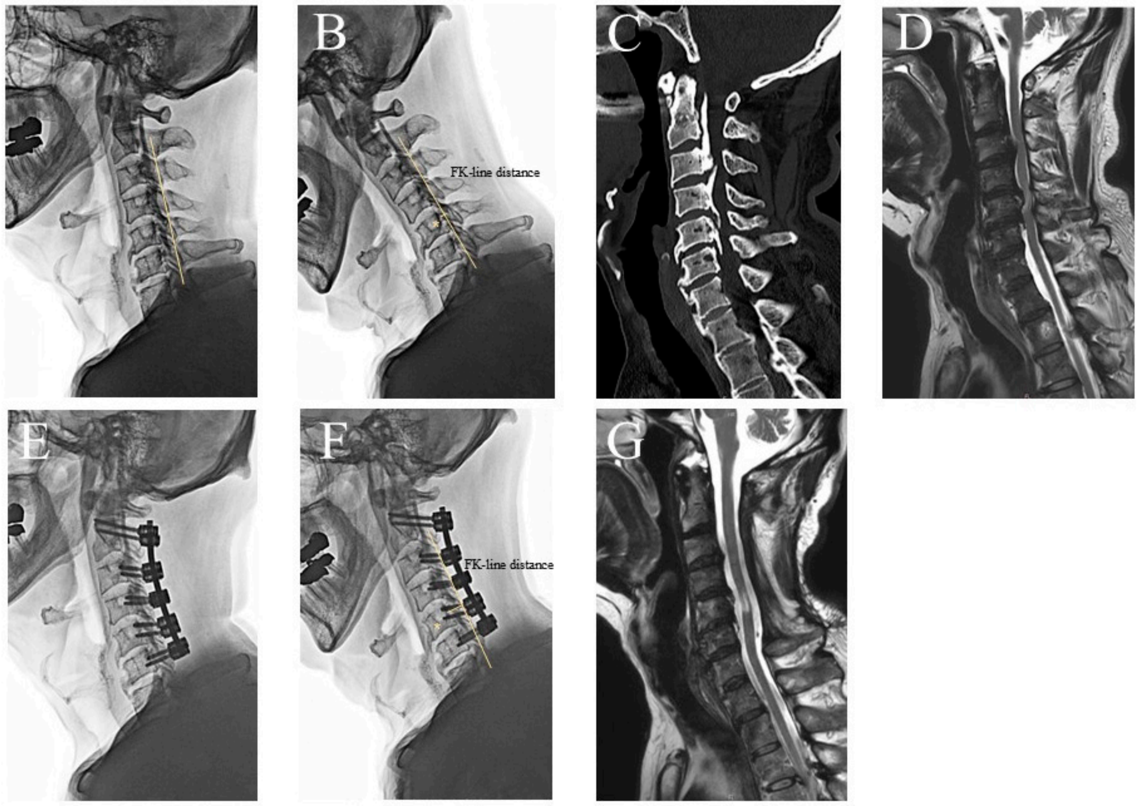

Supplementary Figure S2. Representative case of LF in a patient with NK-line (−) and FK-line (−).

A 73-year-old man with cervical OPLL from C2 to C6 presented with bilateral numbness and weakness in all extremities. (A) Preoperative neutral radiograph shows slightly kyphotic alignment with a negative K-line. (B) Flexion radiograph demonstrates a negative FK-line with a FK-line distance of 1.5 mm (asterisk). (C) Sagittal CT reveals mixed-type OPLL involving C2–C6. (D) T2-weighted MRI confirms severe cord compression and intramedullary signal change at C4–C5. (E) Postoperative neutral radiograph following LF from C2 to C6 shows improved cervical alignment. (F) Postoperative flexion radiograph shows an increased FK-line distance of 10.2 mm (asterisk). (G) Postoperative MRI confirms adequate spinal cord decompression, though residual signal change remains. The JOA score improved from 12 to 16 at 2-year follow-up (recovery rate: 80%). CT = computed tomography; FK-line = flexion K-line; JOA = Japanese Orthopedic Association; LF = laminectomy with fusion; MRI = magnetic resonance imaging; OPLL = ossification of the posterior longitudinal ligament.
